# Association Between Internet Addiction and the Risk of Musculoskeletal Pain in Chinese College Freshmen – A Cross-Sectional Study

**DOI:** 10.3389/fpsyg.2019.01959

**Published:** 2019-09-03

**Authors:** Guang Yang, Jianhua Cao, Yingke Li, Peng Cheng, Bin Liu, Zongji Hao, Hui Yao, Dongzhe Shi, Li Peng, Liya Guo, Zhongyu Ren

**Affiliations:** ^1^School of Physical Education, Chinese Center of Exercise Epidemiology, Northeast Normal University, Changchun, China; ^2^College of Physical Education, Key Laboratory of Physical Fitness Evaluation and Motor Function Monitoring, General Administration of Sport of China, Institute of Sports Science, Southwest University, Chongqing, China; ^3^College of Physical Education, Chongqing Nursing Vocational College, Chongqing, China; ^4^Xinhua College of Sun Yat-sen University, Guangzhou, China

**Keywords:** internet addiction, musculoskeletal pain, Chinese, college freshmen, cross-sectional study

## Abstract

**Background:**

It is well established that increased internet use is related to an increased risk of musculoskeletal pain among adolescents. The relationship between internet addiction (IA), a unique condition involving severe internet overuse, and musculoskeletal pain has, however, not been reported. This study aimed to investigate the association between IA and the risk of musculoskeletal pain among Chinese college students.

**Methods:**

A cross-sectional study was conducted among 4211 Chinese college freshmen. IA status was evaluated using the 20-item Young’s Internet Addiction Test (IAT). IA was defined as internet addiction score ≥50 points. Musculoskeletal pain was assessed using a self-reported questionnaire. Multiple logistic regression analysis was performed to determine association between IA categories (normal, mild, and moderate-to-severe) and musculoskeletal pain.

**Results:**

Among all participants; neck, shoulder, elbow, wrist/hand, and low back and waist pain was reported by 29.2, 33.9, 3.8, 7.9, and 27.9%, respectively. The prevalence of IA was 17.4%. After adjusting for potential confounders, the results showed significant differences in the risk of musculoskeletal pain among different IA categories. The odds ratios (ORs) and 95% confidence intervals (CI) for neck pain with IA categories were 1.000 (reference), 1.451 (1.221, 1.725), and 1.994 (1.608, 2.473), respectively (*P* for trends: < 0.001). For shoulder pain, these were 1.000 (reference), 1.520 (1.287, 1.795), and 2.057 (1.664, 2.542), respectively (*P* for trends: < 0.001). For elbow pain, ORs (95% CIs) were 1.000 (reference), 1.627 (1.016, 2.605), and 2.341 (1.382, 3.968), respectively (*P* for trends: 0.001). Those for wrist/hand pain were 1.000 (reference), 1.508 (1.104, 2.060), and 2.236 (1.561, 3.202), respectively (*P* for trends: < 0.001). For low back and waist pain with severe IA categories, these were 1.000 (reference), 1.635 (1.368, 1.955), and 2.261 (1.813, 2.819), respectively (*P* for trends: < 0.001).

**Conclusion:**

This cross-sectional study showed that severe IA was associated with a higher risk of musculoskeletal pain in Chinese college freshmen. In future research, it will be necessary to explore causality regarding this relationship using interventional studies.

## Introduction

Attending university for the first time is an important life event. The unfamiliar university life requires having to negotiate a new social world while accomplishing significant academic tasks. In contrast to high school students, first-year university students have ample time to either spend with friends or use on the internet. According to data from the China Internet Network Information Center, as of November 2, 2014, 649 million people had accessed the internet. Of these, one-fourth were school and college students (Chi et al., 2016). Due to this rapid growth in internet users, problem with youth internet addiction (IA) has drawn strongly, the attention of researchers. IA is similar to nicotine, alcohol, or drug addiction. It is defined as “a psychological dependence on the Internet, regardless of the activity, once logged on” (Jj, 1998). In Japan, it is estimated that 9.1% of college students have IA (Sato, 2006). In the US, approximately 12% of college students experience symptoms of IA (Jelenchick et al., 2012). In contrast to Western college students, the prevalence of IA in Chinese college students was found to be higher, at 15.2% (Chi et al., 2016). It is well known that IA contributes to the onset of depression (Younes et al., 2016), insomnia (Younes et al., 2016), anxiety (Younes et al., 2016), and poor sleep quality (Zhang et al., 2017). Furthermore, accumulating evidence has shown that excessive internet use (in duration and frequency) can lead to musculoskeletal pain (Ayanniyi et al., 2010; Hellstrom et al., 2015; Dol, 2016; Yang et al., 2017; Borhany et al., 2018). This is because of having to remain in a fixed position over long periods; subsequently, more pressure is placed on several parts of the body involved. Because excessive internet use, including in frequency and duration, is involved in smartphone addiction, it is unclear whether IA would be associated with musculoskeletal pain among adolescents. We hypothesized that IA has potential of adverse effect of musculoskeletal pain (Wu et al., 2016). To our knowledge, a few studies have assessed that IA and musculoskeletal discomfort. One cross-sectional study showed that college students with IA have significantly higher levels of physical fatigue than college students without IA (Bachleda and Darhiri, 2018). Another study reported that IA was positively associated with musculoskeletal pain in adolescents (Tülay and Ejder, 2018). However, in those studies, many confounding factors were not considered and the results have not suggested a significant relationship between IA and musculoskeletal pain.

Because musculoskeletal pain is a leading cause of poor health-related quality of life and chronic disease morbidity (Tüzün, 2007), we designed a cross-sectional study to examine possible relationships between IA and risk of musculoskeletal pain in a large Chinese college freshmen.

## Materials and Methods

### Study Participants

The Southwest University Physical Fitness and Health cohort study is a prospective ongoing study to assess the association between physical fitness and health status of college students at Southwest University (Chongqing, Southwest China). Southwest University has approximately 53,000 full-time students and offers an extensive set of academic disciplines.

In 2018 (baseline period), approximately 4258 students were randomly selected from 35 schools/colleges. We invited all the randomly selected participants (*n* = 4258) to attend a physical fitness program in order to participate in the study. Written informed consent were obtained from participants and their parents, or from the legal guardians for those aged < 16 years. We excluded participants who did not complete the sleep duration assessment (*n* = 4) and participants with missing information on internet and smartphone use (*n* = 43). After these exclusions, this study included a total of 4211 participants (male: 1428) with an age range of 16–24 (mean 18.2, standard deviation [SD] 0.7) years. Ethics approval was obtained from the Institutional Review Board of the College of Physical Education of Southwest University.

### Assessment of IA

Assessment of IA was obtained using the IAT Test, which covers an individual’s Internet use habits, their thoughts about the Internet, as well as the influence of Internet use on their lives (such as compulsive use, withdrawal, related problems at school, work, or sleep). The IAT is a 20-item test with responses ranging from 1 to 5 (with possible sum of scores from 20 to 100). Higher scores represent a more severe state of IA. The following cut-off points were used to distinguish between internet usage categories: normal (0–30 points), mild (31–49 points), and moderate to severe (50–100 points). IA was defined as an IAT score ≥ 50. The reliability and validity of the Chinese version of IAT were described in previous studies (Li et al., 2015; Wu et al., 2016). In the current study, Cronbach’s α for this test was 0.907.

### Assessment of Musculoskeletal Pain

The mNMQ was used to examine pain in six body regions (Crawford, 2007; Yang et al., 2017). The body parts measured by the mNMQ include the neck, shoulders, elbows, wrists or hands, low back, and waist. Each question has a “yes” or “no” response, with a yes denoting relevant pain in that body region.

### Relevant Covariates

Demographic variables included sex, age (continuous variable), single child (yes or no), father’s educational level (senior high school or less, college, or postgraduate), mother’s educational level (senior high school or less, college, or postgraduate), and parents’ marital status (married, widowed, or divorced). Lifestyle factors included smoking status (never, occasionally, or regularly), drinking status (never, occasionally, or regularly), sleep duration (6–8 h/day or not), and sleep quality (good or not). Demographic variables and lifestyle factors were assessed via a self-administered questionnaire. Levels of PA were assessed by the International Physical Activity Questionnaire (Craig et al., 2003). Total weekly PA was calculated by METs × h/week and categorized into two: ≥ 23 MET⋅h⋅week^–1^ or not (Ishikawa-Takata and Tabata, 2007). Depressive symptoms were assessed according to the Chinese version of the Self-Rating Depression Scale (Peng et al., 2013). This scale include 20 items, which are defined as either positive or negative. The participant is required to score each item on a scale of 1–4. The sum of scores for the 20 items range from 20 to 80, with greater values indicating increased severity of depression. In the present study, a total of score > 50 indicates depressive symptoms (Zung, 1965; Xu et al., 2004). The Cronbach α coefficient for the scale is 0.740. Internet and smartphone usage times were separately assessed with the following questions: “How many hours per day do you spend on the computer or smartphone?” We separately grouped the participants into five categories: 0–1 h/day, 1–3 h/day, 3–5 h/day, 5–10 h/day, and >10 h/day.

### Statistical Analysis

All categorical variables were presented as proportions and were compared by logistic regression analysis. Each body part with musculoskeletal pain was used as a dependent variable and categories of IA were used as independent variables. Multiple logistic regression analysis was also used to examine the relationship between categories of IA and each body part with musculoskeletal pain. Model 1 was the crude univariate model; Model 2 was adjusted for sex and age (≤17, 18, 19, and ≥20 years); Model 3 was additionally adjusted for demographic variables and lifestyle factors, as outlined above. Significance was set at *P* < 0.05 for two-sided tests. All tests were performed using IBM SPSS Statistics 24.0 software (IBM SPSS Inc., Chicago, IL, United States).

## Results

The participants’ characteristics according to IA categories were adjusted for sex and age and are presented in Table 1. Parental educational level was lower in participants with severe IA for father (*P* for trend: 0.011 for senior high school or less and 0.043 for college or postgraduate) and mother (0.001, 0.006, respectively). Participants with severe IA reported a higher frequency of occasional drinking, higher PA level (≥ 23 MET⋅h⋅week^–1^), poor sleep quality, and more depressive symptoms (all *P* for trends: < 0.001). Participants with severe IA also had high use of internet (3–5 h/day) and smartphone (3–5, 5–10, and >10 h/day) (all *P* for trend: < 0.001). No other significant differences were observed across categories of IA.

**TABLE 1:** Sex- and age-adjusted participants’ characteristics according to IA level.

***N* = 4211**	**IA level**			
		
	**Normal group (*n* = 1127)**	**Mild group (*n* = 2351)**	**Moderate and severe group (*n* = 733)**	***P* for trend^1^**
**Demographic characteristics**				–
Sex (female)	64.5	66.1	68.5	0.066
Age, %				
≤17 years	12.5	12.6	9.5	0.073
18 years	63.8	64.9	66.0	0.343
19 years	19.6	18.5	20.2	0.804
≥20 years	4.1	3.9	4.2	0.863
Only one child, %	52.6	49.8	51.3	0.565
Father education, %				
Senior high school or less	63.3	70.7	67.7	**0.011**
College	33.5	26.6	30.4	**0.043**
Mother education, %				
Senior high school or less	69.6	76.4	75.7	**0.001**
College	28.0	22.0	23.3	**0.006**
Parent’s marital status, %				
Married	89.4	88.6	88.8	0.690
Widowed	8.2	8.9	8.9	0.560
Divorced	2.5	2.4	2.3	0.810
**Lifestyle factors**				
Smoking status, %				
Regularly	1.0	0.6	0.7	0.443
Occasionally	3.0	2.6	2.0	0.298
Drinking status, %				
Regularly	0.7	0.6	1.1	0.400
Occasionally	39.4	46.4	47.5	**< 0.001**
PA, MET⋅h⋅week^–1^ (≥23)	84.1	80.3	74.5	**< 0.001**
Breakfast frequency				
Everyday	69.1	59.9	51.8	**< 0.001**
Occasionally	30.0	39.6	46.7	**< 0.001**
Internet use duration, %				
0–1 h/day	72.7	71.4	70.4	0.187
1–3 h/day	24.4	25.2	23.2	0.801
3–5 h/day	1.9	2.7	4.6	**< 0.001**
5–10 h/day	0.8	0.6	1.6	0.096
>10 h/day	0.3	0.1	0.1	0.432
Smartphone use duration, %				
0–1 h/day	10.8	6.8	5.9	**< 0.001**
1–3 h/day	49.9	43.1	33.7	**< 0.001**
3–5 h/day	22.0	29.5	31.4	**< 0.001**
5–10 h/day	15.4	17.1	23.9	**< 0.001**
>10 h	2.0	3.5	5.2	**< 0.001**
Sleep duration (6–8 h), %	89.9	91.4	89.1	0.743
Good sleep quality, %	93.8	87.1	77.1	**< 0.001**
Depressive symptoms (SDS ≥ 50), %	0.2	1.4	3.7	**< 0.001**

*^1^*P* for trends were assessed using multivariate logistic regression analyses. Significance of bold values is *P* < 0.05.*

Among all participants, neck, shoulder, elbow, wrist/hand, and low back and waist pain was reported by 29.2, 33.9, 3.8, 7.9, and 27.9%, respectively. Table 2 shows the significant relationships between IA and risk of musculoskeletal pain in different body parts in the multivariate logistic regression models. The ORs (95% CIs) for neck pain in each IA category (normal, mild, and moderate to severe) in Model 3 were 1.00 (reference), 1.451 (1.221, 1.725), and 1.994 (1.608, 2.473), respectively (*P* for trends: < 0.001). The ORs (95% CIs) for shoulder pain in each IA group in Model 3 were 1.00 (reference), 1.520 (1.287, 1.795), and 2.057 (1.664, 2.542), respectively (*P* for trends: < 0.001). The ORs (95% CIs) for elbow pain in each IA group in Model 3 were 1.00 (reference), 1.627 (1.016, 2.605), and 2.341 (1.382, 3.968), respectively (*P* for trends: < 0.001). The ORs (95% CIs) for wrist/hand pain in each IA group in Model 3 were 1.00 (reference), 1.508 (1.104, 2.060), and 2.236 (1.561, 3.202), respectively (*P* for trend: < 0.001). The ORs (95% CIs) for low back and waist pain in each IA group in Model 3 were 1.00 (reference), 1.635 (1.368, 1.955), and 2.261 (1.813, 2.819), respectively (*P* for trends: < 0.001).

**TABLE 2:** Adjusted relationships between IA and the risk of musculoskeletal discomfort in different body parts.

	**Total sample (*n* = 4211)**	**Number of case**	**Model 1^a^**	**Model 2^b^**	**Model 3^c^**
**Neck**					
Normal group	**1127**	237	1.000 (reference)^d^	1.000 (reference)	1.000 (reference)
Mild group	**2351**	700	1.592 (1.345, 1.884)	1.588 (1.341, 1.881)	1.451 (1.221, 1.725)
Moderate and severe group	**733**	293	2.501 (2.035, 3.072)	2.474 (2.011, 3.044)	1.994 (1.608, 2.473)
***P* for trend^e^**	–	–	**<0.001**	**<0.001**	**<0.001**
**Shoulders**					
Normal group	**1127**	280	1.000 (reference)	1.000 (reference)	1.000 (reference)
Mild group	**2351**	818	1.614 (1.376, 1.893)	1.624 (1.379, 1.911)	1.520 (1.287, 1.795)
Moderate and severe group	**733**	329	2.463 (2.020, 3.005)	2.476 (2.020, 3.036)	2.057 (1.664, 2.542)
***P* for trend^e^**	–	–	**<0.001**	**<0.001**	**<0.001**
**Elbows**					
Normal group	**1127**	23	1.000 (reference)	1.000 (reference)	1.000 (reference)
Mild group	**2351**	91	1.933 (1.216, 3.071)	1.932 (1.216, 3.070)	1.627 (1.016, 2.605)
Moderate and severe group	**733**	48	3.364 (2.028, 5.579)	3.334 (2.009, 5.532)	2.341 (1.382, 3.968)
***P* for trend^e^**	–	–	**<0.001**	**<0.001**	**0.001**
**Wrists/hands**					
Normal group	**1127**	56	1.000 (reference)	1.000 (reference)	1.000 (reference)
Mild group	**2351**	185	1.633 (1.201, 2.222)	1.627 (1.196, 2.214)	1.508 (1.104, 2.060)
Moderate and severe group	**733**	91	2.711 (1.916, 3.835)	2.681 (1.894, 3.795)	2.236 (1.561, 3.202)
***P* for trend^e^**	–	–	**<0.001**	**<0.001**	**<0.001**
**Low back and waist**					
Normal group	**1127**	211	1.000 (reference)	1.000 (reference)	1.000 (reference)
Mild group	**2351**	679	1.763 (1.481, 2.099)	1.762 (1.478, 2.100)	1.635 (1.368, 1.955)
Moderate and severe group	**733**	284	2.746 (2.224, 3.391)	2.718 (2.197, 3.362)	2.261 (1.813, 2.819)
***P* for trend^e^**	–	–	**<0.001**	**<0.001**	**<0.001**

*^*a*^Model 1: Crude; ^*b*^Model 2: Adjusted for sex, age (≤17 years, 18 years, 19 years, ≥20 years); ^*c*^Model 3: Additionally adjusted for only on child (yes or no), father education (senior high school or less, college or undergraduate), mother education (senior high school or less, college or undergraduate), parent’s marital status (married, widowed, divorced), smoking status (regularly, occasionally, never), drinking status (regularly, occasionally, never), PA (≥23 MET⋅h⋅week^–1^ or not), sleep duration (6–8 h or not), good sleep quality (yes or no), depressive symptoms (SDS ≥ 50 or not), internet use duration (0–1, 1–3, 3–5, 5–10, >10 h), and smartphone use duration (0–1, 1–3, 3–5, 5–10, >10 h); ^*d*^Adjusted data are expressed as odds ratio (95% confidence intervals); ^*e*^*P* for trend were obtained using multivariate logistic regression analyses. Significance of bold values is *P* < 0.05.*

## Discussion

A cross-sectional study was conducted in Chinese college freshmen to assess the relationship between IA and risk of musculoskeletal pain in different body parts. Multivariate logistic analyses showed that severe IA was significantly and independently related with higher risk of musculoskeletal pain in different body parts after adjusting for potential confounders.

Currently, the relationship between greater internet use and increased risk of musculoskeletal pain is widely accepted (Ayanniyi et al., 2010; Hellstrom et al., 2015; Dol, 2016; Yang et al., 2017; Borhany et al., 2018). However, to the best of our knowledge, the relationship between IA and the risk of musculoskeletal pain had not been confirmed. Because the current study confirmed previous findings on IA status and the use of internet and smartphones (Haug et al., 2015; Liu et al., 2016), we conclude that IA is significantly related to an increased risk of musculoskeletal pain in Chinese college freshmen.

Although the exact etiology of the association between IA and musculoskeletal pain is not yet known, we explored two possible reasons. A plausible explanation is that the association is mediated by daily internet time. It has been hypothesized that participants with IA may use the internet for longer, on a daily basis, which subsequently increases the risk of musculoskeletal pain. During internet or smartphone use, such as while chatting with a friend by text messaging or playing games online, users often remain in a fixed position while gripping their smartphones for an extended period of time with wrists extended and pronated, elbows flexed, and head down. These poor postural habits could lead to strain on the muscles, tendons, and disks, which leads to neck, shoulder, elbow, and wrist/hand pain (Kim et al., 2015). Longer daily internet usage was an emerging risk factor for low back and waist pain due to sedentary behavior. Prolonged sedentary and otherwise incorrect posture are also considered to be important risk factors in the development of low back pain (Balague et al., 1999).

Alternatively, unhealthy dietary behaviors could also explain our findings. The present study revealed that severe IA status was also associated with skipping of breakfast and less PA (Table 1). A population-based study has shown that, compared to regular breakfast consumers, adolescent breakfast skippers are often deficient in vitamin D (Mielgo-Ayuso et al., 2017). For Chinese college students, dairy products are important breakfast foods, and the lack of this food source as well as reduced exposure to sunlight can contribute to low vitamin D levels (Ghai et al., 2015). Interestingly, vitamin D deficiency can lead to selective alterations in target innervation, resulting in presumptive nociceptor hyperinnervation of skeletal muscle, which in turn is likely to contribute to muscular hypersensitivity and pain.

In the present study, other potential confounding factors that might influence the results were analyzed, including sex, age, single child status, educational level of each parent, parents’ marital status, smoking status, drinking status, PA, sleep duration, sleep quality, and depressive symptoms. Even after adjusting for these potential confounders, a significant association between IA and musculoskeletal pain remained, indicating that this association was independent.

The effect of internet on people’s lives is controversial (Musetti et al., 2017). Several authors thought that internet use should not be seen as a mere instrumental action to achieve a goal (which could be functional or dysfunctional), however, rather, they propose treating internet use as an action situated in the digital context, as part of a system with a proper structure and rules; undoubtedly, mobile or wearable devices such as smartphones are part of people’s daily engagements today (Musetti and Corsano, 2018). Furthermore, they allow for continuous online access, which shapes the course of people’s daily activities and interactions (Smart, 2017), but also that they result in people being pathologically dependent on the internet. These subsequently lead to the onset of internet-related symptomatology (Schimmenti et al., 2014b; Billieux et al., 2015; Musetti et al., 2018). Consequently, we considered that the identification of a diagnostic category concerning internet-related symptomatology is necessary (Schimmenti et al., 2014a). So far, internet-related symptomatology mainly focuses on IA and Internet Gaming Disorder. However, it is rather clear that the diagnostic category of IA is more consistent with research findings than that for Internet Gaming Disorder. Furthermore, the diagnosis of IA would be even more meaningful clinically (Schimmenti et al., 2014a). Future studies to structure a more innovative, integrated, and comprehensive model and to formulate precise diagnostic criteria for defining IA status should be conducted (Musetti et al., 2016). This is because, these studies may provide evidence regarding identifying IA as a pathological condition that can be used by physicians. The present study suggests that not only excessive internet duration and frequency, but severe status of IA needs also to be taken into consideration for reducing the risk of musculoskeletal pain.

There are several limitations to our study. First, the present work was a population-based cross-sectional study and therefore the causative link between IA and musculoskeletal pain could not be established. Secondly, although a cut-off point (IAT score of 50) was used to define having moderate-to-severe IA, we were unable to establish a clinical diagnosis to determine morbidity for each participant with IA. Finally, the results may not be representative of the Chinese general college freshmen; therefore, further investigations with larger sample sizes are needed to confirm our findings.

## Conclusion

This cross-sectional study indicates that severe IA is associated with higher risk of musculoskeletal pain. In future research, it will be necessary to explore the causative links between IA and musculoskeletal pain with prospective cohort or other interventional studies.

## Data Availability

The datasets generated for this study are available on request to the corresponding author.

## Author Contributions

ZR, LP, and LG conceived and designed the experiments. JC, YL, PC, BL, ZH, HY, and DS performed the experiments and conducted the data collection. GY and JC analyzed the data. PC and ZR contributed to the reagents, materials, and analysis tools. GY and JC wrote the manuscript. All authors contributed to the manuscript revision, read and approved the submitted version.

## Conflict of Interest Statement

The authors declare that the research was conducted in the absence of any commercial or financial relationships that could be construed as a potential conflict of interest.

## Abbreviations

CI: confidence interval
IA: internet addiction
IAT: Internet Addiction Test
MET: metabolic equivalents
mNMQ: modified nordic musculoskeletal questionnaire
OR: odds ratio
PA: physical activity.
